# The C18:3n6/C22:4n6 ratio is a good lipid marker of chronic kidney disease (CKD) progression

**DOI:** 10.1186/s12944-020-01258-y

**Published:** 2020-04-17

**Authors:** Małgorzata Szczuko, Małgorzata Kaczkan, Sylwia Małgorzewicz, Przemysław Rutkowski, Alicja Dębska-Ślizień, Ewa Stachowska

**Affiliations:** 1 Department of Human Nutrition and Metabolomics, Pomeranian Medical University in Szczecin, Szczecin, Poland; 2 Department of Clinical Nutrition and Dietetics, Medical University of Gdańsk, Gdańsk, Poland; 3 Department of General Nursery, Medical University of Gdańsk and Diaverum Hemodialysis Unit, Gdańsk, Poland; 4 Department of Nephrology, Transplantology and Internal Medicine, Medical University of Gdańsk, Gdańsk, Poland

**Keywords:** Fatty acid, Chronic kidney disease, Adrenic acid, Docosatetraenoate acid, Gamma linoleic acid

## Abstract

**Background:**

Chronic kidney disease (CKD) is a major challenge for public health due to increased risk of cardiovascular diseases (CVD) and premature death. The aim of this study was to determine the clinical picture of FA and the course of the pathophysiological mechanisms of CKD.

**Methods:**

The study involved 149 patients with CKD and a control group including 43 people. Fatty acid profiles were investigated using gas chromatography. A total of 30 fatty acids and their derivatives were identified and quantified. The omega3, omega6, SFA, MUFA, and PUFA fatty acid contents were calculated. The correlation matrix was obtained for parameters relating to patients with CKD vs. FA, taking patients’ sex into consideration. The index C18:3n6/C22:4n6 was calculated according to the length of the treatment. Statistica 12.0 software (Tulsa, Oklahoma, USA) was used for the statistical analyses.

**Results:**

The results showed decreased levels of total PUFA and increased concentrations of MUFA, including the activation of the palmitic and oleic acid pathway. An increase in the levels of n-6 9C22: 4n6 family fatty acids in all the patients and a reduction in the n-3 family (EPA, DHA) were observed. C18:3n6 was negatively correlated and C22:4n6 was positively correlated with the duration of the treatment. The index C18:3n6/C22:4n6 was defined as a new marker in the progression of the disease. Moreover, the index C18:3n6/ C22:4n6 was drastically decreased in later period. Nervonic acid was higher in the CKD group. In the group of men with CKD, there was a negative correlation between the excretion of K+, anthropometric measurements, and the levels of EPA and DHA.

**Conclusions:**

The course of inflammation in CKD occurs through the decrease in PUFA and the synthesis of MUFA. The dominating cascade of changes is the elongation of GLA-C18:3n6 into DGLA-C20:3n6 and AA-C20:4n6. As CKD progresses, along with worsening anthropometrical parameters and increased secretion of potassium, the activity of Ʌ6-desaturase decreases, reducing the synthesis of EPA and DHA. The synthesis of AdA-C22:4n6 increases and the ratio C18:3n6/C22:4n6 drastically decreases after 5 years. This parameter can be used to diagnose disease progression.

## Highlights

We present a clinical picture of free fatty acid (FFA) and the course of the pathophysiological mechanisms of chronic kidney disease (CKD).

The results showed decreased levels of total polyunsaturated fatty *acids* (PUFA) and increased concentrations of monounsaturated fatty *acids* (MUFA), including the activation of the palmitic and oleic acid pathway.

The index C18:3n6/C22:4n6 was stable for up to 5 years of treatment but drastically decreased in the later period. This parameter can be used to diagnose disease progression.

The dominating cascade of changes is the elongation of GLA-C18:3n6 into DGLA-C20:3n6 and AA-C20:4n6.

## Background

Chronic kidney disease (CKD) is a major challenge for public health due to its high incidence, common progression to end-stage renal disease (ESRD), and the higher risk of cardiovascular disease (CVD) and premature death [1, 2]. The profile of fatty acids (FA) changes due to diet, but it also changes in some diseases, such as debilitating diseases that lead to malnutrition and increased catabolism (cachexia) [3].

Fatty acids (FA) are an important source of energy but also act as signal molecules in various cellular processes. Hirasawa et al. found a connection between the secretion of intestinal incretin peptides and an increased level of free insulin [4]. It was shown that the receptor coupled with G protein, GPR120, which is abundantly exprimated in intestines, acts as a receptor for unsaturated long-chained FA. Therefore, the stimulation of GPR120 by FA promotes the secretion of GLP-1 (the strongest insulinotropic incretin) in vitro and in vivo, thus increasing free insulin. So far, the studies on FA have suggested that the level of FA in blood plasma mainly results from the diet. However, our studies show that in patients with CKD the concentration of FA in plasma is dependent on the stage of the disease and the catabolism of adipose tissue, which takes part in the progression of inflammation and is the cause of CKD development. Such a conclusion was also drawn by other researchers: Afshinnia et al. connected an increased level of saturated FA C16:0-C20:0 with impaired β-oxidation of FA and reverse partitioning/binding into complex lipids as mechanisms facilitating changes in lipid metabolism which characterize progressing CKD [5]. Inflammatory stress enhances kidney damage through the activation of the CD36 pathway. This mechanism may occur in obese people with chronic inflammation, which makes them susceptible to CKD [6]. It seems that increased fat content in liver is linked to increased occurrence of mild and heavy albuminuria and decreased glomerular filtration rate (GFR). Therefore, non-alcoholic fatty liver disease (NAFLD) may be a risk factor for diabetic nephropathy (DN), and increased fat content in liver may be connected to higher DN risk [7]. However, these results are not consistent among various researchers. Zhan et al., when examining 413 patients with type 2 diabetes (T2DM) and with or without NAFLD, did not observe this connection between NAFLD and DN frequency in patients with T2DM [8]. In contradiction to that, two cohort studies performed by Targher et al. showed that renal disfunction was significantly more frequent in patients with NAFLD [9, 10]. The authors of the study already compared the FA profile of CKD patients with the profile of patients with Metabolic Syndrome (MetS); it was observed that there had been an increase in the amounts of all FAs in plasma in the CKD group, especially in the case of palmitic acid (C16:0) and the derivatives of stearic acid (C18:0), higher than in the MetS patients; this may result from the decomposition of adipose tissue and the progressing devastation of the organism [11]. Furthermore, stearoyl-CoA desaturase (SCD) is a reticulum enzyme that catalyzes of monounsaturated fatty acids from saturated fatty acids [12]. The preferred substrates are palmitoyl- and stearoyl-CoA, which get converted into palmitoleoyl- and oleoyl-CoA. These products are the most widespread monounsaturated fatty acids (MUFA) in the lipids, including phospholipids, triglycerides, cholesteryl esters, wax esters, and alkyldiacylglycerols [13]. Given the multiple roles of MUFA, variation in SCD activity in mammals would be expected to have an different effects on metabolism and physiology, including differentiation, insulin sensitivity, metabolic rate, adiposity, obesity, atherosclerosis and cancer [12]. It is also likely that both diseases such as atypical hemolytic-uremic syndrome (aHUS) and membranoproliferative glomerulonephritis (MPGN) associated with rather acute renal failure under the influence of infection (*E. coli* serotype c157: H7, Shigella, Yersinia, Streptococus, lupus erythematosus) depending on the immune mechanism with the participation of the C3 complement component may also be one of the mechanisms of FA synthesis change [14–16]. Because in our research we had a group of subjects older in the majority and there were no cases of acute renal failure, this mechanism is probably considered to be less significant but possible. Moreover, in MetS, auxiliary metabolic pathways are activated for oleic acid and cause the simultaneous inhibition of EPA and DHA synthesis from ALA, whereas in the CKD group, we observe increased synthesis of EPA and DHA. The higher increase of nervonic acid (C24:1) in CKD suggests a higher degree of demyelination and loss of anons [11].

The aim of the present study was to investigate the influence of the duration of the disease on the clinical picture of FA, which can help in understanding the mechanisms of CKD pathophysiology and in adjusting therapy to be better for patients.

## Methods

### Participants in the study

All laboratory measurements characterizing the patients with CKD were performed at Central Laboratory Medical University of Gdańsk under rigorous quality control. The studies concerning FA contents were carried out at the Department of Human Nutrition and Metabolomics, Pomeranian Medical University in Szczecin.

This study was an ongoing multicentre prospective cohort study. It involved 192 Caucasian people from the central European region. The first test group comprised patients with chronic kidney disease (CKD). These were 62 women at the age of 65.9 ± 13.98, with an average body weight of 69.33 ± 18.47 kg,, and 87 men at the age of 63.4 ± 15.9, with an average weight of 76.6 ± 15.97 kg. Other parameters characteristic of the disease profile are presented in Table 1.

**Table 1:** Characteristics of the patients with chronic kidney disease (CKD) divided into sexes

Characteristic	Women *n* = 62 Avg + SD	Men *n* = 87 Avg + SD	Significance of differences
Age ([years)]	65.88 ± 13.98	63.40 ± 15.90	NS
Weight ([kg)]	69.33 ± 18.47	76.60 ± 15.97	0.0479
BMI [kg/m2]	24.81 ± 6.14	25.28 ± 6.6	NS
Ca [mg/dl]	8.66 ± 0.97	8.47 ± 0.79	NS
I PHOS [mg/dl]	5.375 ± 1.36	5.03 ± 1.26	NS
iPTH [pg/ml]	705.8 ± 663.9	416.2 ± 364.7	0.0097
CREA [mg/dl]	7.49 ± 2.07	8.41 ± 2.35	NS
HGB [g/dl]	10.67 ± 1.11	10.74 ± 1.37	NS
%TSAT [%]	30.57 ± 15.22	29.24 ± 13.29	NS
FER [ug/l]	954,9 ± 739.5	689.2 ± 616.9	0.0000
ALB [g/l]	34.56 ± 4.31	35.80 ± 3.92	NS
K [mEq/L]	5.347 ± 0.64	5.261 ± 0.63	NS
Treatment duration [months]	62.42 ± 79.38	46.29 ± 52.01	NS
Body surface [m2]	1.727 ± 0.21	1.846 ± 0.186	0.0074
Kt/V	1.716 ± 0.31	1.579 ± 0.33	0.0136
nPCR [g/kg/day]	1.071 ± 0.24	1.09 ± 0.267	NS
Ca x P [mg2/dl2]	47.18 ± 11.63	42.096 ± 12.457	NS

Criteria for starting renal replacement therapy:

1. Clinical

- if the pacjent has symptoms of uremia, conduction, uncontrolled hypertension or signs of malnutrition, hyperkalemia, severe metabolic acidosis,

2. Biochemical

- eGFR < 10 ml/min or < 15 in people with diabetes, urea in serum 160–200 mg/dl

The decision is always made individually.

The second group, i.e., the control group, comprised 43 healthy people, including 19 women at the age of 53.21 ± 4.16 and with an average weight of 59.39 ± 4.72 kg and 24 men at the age of 57.10 ± 4.38 and with an average weight of 76.42 ± 5.27 kg. In the control group, all women and men had appropriate body weight with respect to height (Table 2).

**Table 2:** Characteristics of the control groups divided into sexes

Characteristic	Women *n* = 15 Avg + SD	Men *n* = 39 Avg + SD	Significance of differences
Age ([years)]	53.21 ± 4.16	57.10 ± 4.38	NS
Weight ([kg)]	59.39 ± 4.72	76.42 ± 5.27	0.0031
BMI [kg/m2]	22.86 ± 1.21	24.31 ± 1.26	NS

### FAME extraction

Blood plasma was obtained from whole-blood samples collected in tubes containing EDTA as an anticoagulant by centrifugation for 10 min at 1200 G. Plasma samples were stored at − 80 °C. Fatty acids were extracted according to the Folch method and analyzed by gas chromatography. A quantity of 0.5 ml of plasma was saponified with 1 ml of 2 mol/L KOH methanolic solution at 70 °C for 20 min and then methylated with 2 ml of 14% boron trifluoride in methanol under the same conditions. Then 2 ml of n-hexane and 10 ml of saturated NaCl solution were added. A quantity of 1 ml of the n-hexane phase was collected for analysis.

### Gas chromatography (GC) anaylsis

The analysis of the fatty acids profile in the plasma was performed using gas chromatography on an Agilent Technologies 7890A GC System, and the compounds were separated using a Capillary GC Column (15 mm × 0.10 mm, 0.10 μm; Supelco, Bellefonte, PA, United States) (SUPELCOWAX™ 10). The chromatographic conditions were as follows: the initial temperature was 40 °C for 0.5 min; it was increased at a rate of 25 °C/min to 195 °C (0 min); next, it was increased at a rate of 3 °C/min to 205 °C (0 min) and then increased at a rate of 8 °C /min to 250 °C for 0.5 min. The total analysis lasted approximately 16.158 min and the gas flow rate was 1 mL/min with hydrogen as the carrier gas. Fatty acids were identified by comparing their retention times with those of commercially available standards. The analysis of the fatty acids content in particular samples was performed using specialist ChemStation Software (Agilent Technologies, UK). Qualitative identification of the fatty acids was performed by comparing retention times to those of the standards, and the quantity of a specific fatty acid was given as a percentage of the fatty acids in the total amount of analysed compounds (excluding the standard—heneicosanoic acid (C21:0)).

The fatty acid content was calculated as follows [17]:

sum of the omega3 fatty acids: (n3) = C18:3n3 + C20:5n3 + C22:5n3 + C22:6n3;

sum of the omega 6 fatty acids: (n6) = C18:2n6 + C18:3n6 + C20:3n6 + C20:4n6 + C22:4n6;

sum of the saturated fatty acids:

(SFAs) = C8:0 + C10:0+ C11:0 + C12:0 + C14:0 + C15:0 + C16:0 + C17:0 + C18:0 + C20:0 + C22:0 + C23:0 + C24:0);

sum of the monounsaturated fatty acids:

(MUFAs) = C14:1n-9 + 16:1n7 + C17:1 + C18:1 + C22:1 + C24:1),

sum of the poly-unsaturated fatty acids:

(PUFAs) = 18:2n6 + C18:3n6 + C18:3n3 + C18:3n6 + C20:3n3 + C20:4n6 + C20:5n3 + C22:4n6 + C22:5n3 + C22:6n3. The UFAs were the sum of the MUFAs and PUFAs.

### Statistical analysis

Statistica 12.0 software (Tulsa, Oklahoma, USA) was used for the statistical analyses, and all results were expressed as mean ± standard deviation. As the distribution in most cases was normal (Shapiro–Wilk Test), parametric *t*-testing was used for comparisons between groups (Men CKD vs. CG; Women CKD vs. CG; Women CKD vs. Men CKD), and *p* ≤ 0.05 was considered statistically significant. The correlation matrix was obtained for the biochemical and anthropometric parameters of patients with CKD vs. FA taking patients’ sex into consideration. In Tables 6 and 7 are presented the correlations of those FA for which there were at least two significant correlations. Next, all the patients with CKD were divided into six groups (of similar size) according to the length of the treatment, and the index C18:3n6/C22:4n6 was calculated to compare the significance of differences between the groups. Due to the multitiude of statistically insignificant resolts, we perfomed a post hoc power analysis. The power of tests which showed no statistical significanse, was below the recomended level of 0,8. On the over hand tests showing statistical significans had an acceptable power reaching as high as 0,82.

## Results

### Participants in the study

The group of patients with CKD varied depending on the sex. The group of women with CKD differed significantly when compared to men with CKD with respect to body weight, intact parathyroid hormone (iPTH), concentrations of creatinine and ferritin, duration of the treatment, body surface, Kt/V (dialysis adequacy, Table 1). In the control groups, the differences between the sexes related only to body weight (Table 2).

### Comparison of FA percentages in plasma

Analyses of the percentages of FA in plasma in the group of men with CKD showed that there are fundamental differences in the concentration of the majority of fatty acids in comparison to the control group (Table 3). The most significant differences in medium-chain FA related to the decreases in C8:0, C11:0, C17:0, C18:0, and C18:2n6 and the increases in C10:0, C15:0, C16:0, C16:1, C18:1n9, and C18:1 t. Also, decreased levels of C20:3n6 C20:5n3, C22:5n3, and C22:6n3 and increased levels of C22:0, C23:0, C22:4n6, and C24:1 were observed. These changes were basically connected with the increased SFA and omega-6 family (C22:4n6) concentrations and the reduction in the index C18:3n6/C22:4n6 in comparison to the control group (Table 3).

**Table 3:** Comparison of the percentages of FFA in the plasma of men with CKD vs. the control group

PARAMETER	MEN (CKD)		CONTROL		p.value
	MEAN	SD	MEAN	SD	
C 8:0 Caprylic acid	0.0073	0.033	0.0611	0.024	*p* < 0.05
C10:0 Capric acid	0.9849	0.624	0.0098	0.020	p < 0.05
C11:0 Undecanoic acid	0.0236	0.015	0.0636	0.021	p < 0.05
C12:0 Lauric acid	0.1460	0.117	0.1556	0.069	NS
C14:0 Myristic acid	1.3045	0.793	1.2106	0.337	NS
C14:1 Myristolenic acid	0.0788	0.072	0.0783	0.077	NS
C15:0 Pentadecanoid acid	0.3474	0.142	0.2779	0.064	p < 0.05
C16:0 Palmitic acid	28.616	1.975	26.598	2.097	p < 0.05
C16:1 Palmitoleic acid	1.8664	0.812	1.4488	0.524	p < 0.05
C17:0 Heptadecanoid acid	0.3413	0.082	0.4520	0.050	p < 0.05
C17:1 cis-10- Heptadecanoid acid	0.1155	0.088	0.1347	0.059	NS
C18:0 Stearic acid	11.469	1.451	13.145	2.073	p < 0.05
C18:1n9 ct Oleic acid	22.933	3.232	19.585	2.435	p < 0.05
C18:1 tans vaccinic acid	2.1048	0.304	1.7612	0.228	p < 0.05
C18:2n6c Linoleic acid	16.856	3.385	20.509	2.009	p < 0.05
C18:3n6 gamma linoleic acid	0.1980	0.090	0.2206	0.093	NS
C18:3n3 linolenic acid	0.6846	0.307	0.5976	0.230	NS
C20:0 Arachidic acid	0.1623	0.051	0.1548	0.083	NS
C22:1/C20:1 cis11- eicosanic acid	0.2369	0.079	0.2209	0.088	NS
C20:2 cis-11-eicodienoic acid	0.2041	0.063	0.1820	0.018	NS
C20:3n6 eicosatrienoic acid	1.0763	0.260	1.3352	0.340	p < 0.05
C20:4n6 Arachidonic acid	5.7957	1.278	6.4857	1.429	NS
C20:5n3 EPA	0.7535	0.466	1.4693	1.193	p < 0.05
C22:0 Behenic acid	0.0531	0.046	0.0148	0.032	p < 0.05
C23:0 tricosanoic acid	0.2310	0.400	0.0555	0.065	p < 0.05
C22:4n6 (docosatetraenoate)	0.3210	0.199	0.1295	0.032	p < 0.05
C22:5w3 (docosapentaenate)	0.5764	0.172	0.6740	0.083	p < 0.05
C24:0 Lignoceric acid	.0.3034	0.852	0.5165	0.882	NS
C22:6n3 DHA	1.912	0.664	2.1039	1.576	p < 0.05
C24:1 Nervonic acid	0.0566	0.075	0	0	p < 0.05
n3	3.8038	1.227	4.8446	1.890	p < 0.05
n6	23.496	4.886	28.680	3.825	p < 0.05
SFA	42.979	7.470	42.712	3.987	NS
MUFA	27.034	5.523	23.228	2.786	p < 0.05
PUFA	27.692	5.524	33.706	3.759	NS
C18:3n6/C22:4n6	0.9261	0.931	1.7037	0.999	p < 0.05

A comparison of the group of women with CKD with the control group showed lower concentrations of SFA, especially C8:0, C11:0, C17:0, C18:0, and C20:0, and lower levels of C17:1, C18 2n6, C20:3n6, C20:4n6, C20:5n3, C22:5n3, and C22:6n3. However, increased percentages were noted in the cases of C10:0, C14:1, C16:1, C18:1n9, C18:1 t, C22:4n6, and C24:0 (Table 4). To summarize, in the group of women with CKD, there was a reduction in the ratio of the n-3 family (EPA, DHA) and a several-fold increase in the n-6 family (C22:4n6) but reductions in C20:3n6 and C20:4n6. Similarly, as in the group of men with CKD, total SFA was unchanged relative to the control group. There was an increase in the total MUFA and drop in the PUFA and a reduced C18:3n6/C22:4n6 ratio in comparison with the control group (Table 4). When comparing men with CKD to women with CKD, there were a few differences in the percentage of FA in plasma which related to higher concentrations of C18:0, C18:1 t, and C20:0 in the male group with CKD and of C18:3n6 in the female group with CKD (Table 5).

**Table 4:** Comparison of the percentages of FFA in the plasma of women with CKD vs. the control group

PARAMETER	WOMEN (CKD)		CONTROL		p.value
	MEAN	SD	MEAN	SD	
C 8:0 Caprylic acid	0.0070	0.022	0.0568	0.026	p < 0.05
C10:0 Capric acid	0.8944	0.638	0.0172	0.022	p < 0.05
C11:0 Undecanoic acid	0.0209	0.013	0.0840	0.022	p < 0.05
C12:0 Lauric acid	0.1570	0.118	0.1473	0.049	NS
C14:0 Myristic acid	1.3306	0.605	1.0995	0.348	NS
C14:1 Myristolenic acid	0.0877	0.063	0.0587	0.044	p < 0.05
C15:0 Pentadecanoid acid	0.3476	0.133	0.2812	0.064	NS
C16:0 Palmitic acid	27.8722	2.781	27.6025	1.579	NS
C16:1 Palmitoleic acid	1.9987	0.678	1.5677	0.371	p < 0.05
C17:0 Heptadecanoid acid	0.3519	0.088	0.4528	0.049	p < 0.05
C17:1 cis-10- Heptadecanoid acid	0.1141	0.102	0.1387	0.065	p < 0.05
C18:0 Stearic acid	10.9025	1.855	12.3402	1.241	p < 0.05
C18:1n9 ct Oleic acid	22.8029	4.134	18.8294	1.103	p < 0.05
C18:1 tans vaccinic acid	1.9982	0.331	1.7374	0.158	p < 0.05
C18:2n6c Linoleic acid	17.8465	2.910	20.5960	1.719	p < 0.05
C18:3n6 gamma linoleic acid	0.2418	0.123	0.2429	0.065	NS
C18:3n3 linolenic acid	0.6727	0.307	0.6145	0.166	NS
C20:0 Arachidic acid	0.1449	0.051	0.1677	0.030	p < 0.05
C22:1/C20:1 cis11- eicosanic acid	0.2247	0.116	0.2180	0.034	NS
C20:2 cis-11-eicodienoic acid	0.1893	0.059	0.2170	0.032	p < 0.05
C20:3n6 eicosatrienoic acid	1.1222	0.294	1.5610	0.423	p < 0.05
C20:4n6 Arachidonic acid	5.9699	1.299	7.5353	1.155	p < 0.05
C20:5n3 EPA	0.7960	0.371	0.9682	0.248	p < 0.05
C22:0 Behenic acid	0.0545	0.041	0.0237	0.039	p < 0.05
C23:0 tricosanoic acid	0.2924	0.547	0.0359	0.044	p < 0.05
C22:4n6 (docosatetraenoate)	0.3470	0.211	0.1338	0.094	p < 0.05
C22:5w3 (docosapentaenate)	0.5866	0.128	0.7574	0.194	p < 0.05
C24:0 Lignoceric acid	0.4644	0.769	0.0000	0.000	p < 0.05
C22:6n3 DHA	2.0641	0.538	2.5156	0.460	p < 0.05
C24:1 Nervonic acid	0.0582	0.068	0.0000	0.000	p < 0.05
n3	3.9500	1.108	4.8556	1.033	p < 0.05
n6	24.8704	4.869	30.0689	5.368	p < 0.05
SFA	42.1493	6.306	42.3087	3.965	NS
MUFA	27.0751	5.744	22.4911	2.249	p < 0.05
PUFA	29.0583	5.268	34.9245	2.365	p < 0.05
C18:3n6/C22:4n6	0.9162	0.686	1.8153	2.563	p < 0.05

**Table 5:** Comparison of the percentages of FFA in the plasma of men with CKD vs. women with CKD

PARAMETER	WOMEN (CKD)		MEN (CKD)		p.value
	MEAN	SD	MEAN	SD	
C 8:0 Caprylic acid	0.0071	0.022	0.0073	0.033	NS
C10:0 Capric acid	0.8944	0.638	0.9849	0.624	NS
C11:0 Undecanoic acid	0.0209	0.13	0.0236	0.015	NS
C12:0 Lauric acid	0.1570	0.118	0.1460	0.117	NS
C14:0 Myristic acid	1.3306	0.605	1.3045	0.793	NS
C14:1 Myristolenic acid	0.0877	0.063	0.0788	0.073	NS
C15:0 Pentadecanoid acid	0.3476	0.133	0.3474	0.142	NS
C16:0 Palmitic acid	27.8720	2.781	28.6156	1.976	NS
C16:1 Palmitoleic acid	1.9987	0.678	1.8664	0.812	NS
C17:0 Heptadecanoid acid	0.3519	0.088	0.3413	0.082	NS
C17:1 cis-10- Heptadecanoid acid	0.1141	0.102	0.1155	0.088	NS
C18:0 Stearic acid	10.9030	1.855	11.4695	1.451	p < 0.05
C18:1n9 ct Oleic acid	22.8030	4.134	22.9331	3.232	NS
C18:1 tans vaccinic acid	1.9982	0.331	2.1048	0.304	p < 0.05
C18:2n6c Linoleic acid	17.8470	2.910	16.8563	3.385	NS
C18:3n6 gamma linoleic acid	0.2418	0.123	0.1980	0.090	p < 0.05
C18:3n3 linolenic acid	0.6727	0.307	0.6846	0.307	NS
C20:0 Arachidic acid	0.1449	0.051	0.1623	0.051	p < 0.05
C22:1/C20:1 cis11- eicosanic acid	0.2247	0.116	0.2369	0.079	NS
C20:2 cis-11-eicodienoic acid	0.1893	0.059	0.2041	0.063	NS
C20:3n6 eicosatrienoic acid	1.1222	0.294	1.0763	0.260	NS
C20:4n6 Arachidonic acid	5.9699	1.299	5.7957	1.278	NS
C20:5n3 EPA	0.7960	0.371	0.7535	0.466	NS
C22:0 Behenic acid	0.0545	0.041	0.0531	0.046	NS
C22:1n9 13 (Erucic acid	0.2412	1.025	0.2797	1.577	NS
C23:0 tricosanoic acid	0.2924	0.547	0.2310	0.400	NS
C22:4n6 (docosatetraenoate)	0.3395	0.223	0.3203	0.205	NS
C22:5w3 (docosapentaenate)	0.5866	0.128	0.5764	0.172	NS
C24:0 Lignoceric acid	0.4644	0.769	0.3034	0.852	NS
C22:6n3 DHA	2.0641	0.538	1.9231	0.660	NS
C24:1 Nervonic acid	0.0582	0.068	0.0573	0.075	NS
n3	3.9500	1.108	3.8038	1.234	NS
n6	24.8700	4.869	23.4958	4.914	NS
SFA	42.1493	6.306	42.9788	7.513	NS
MUFA	27.0751	5.744	27.0343	5.555	NS
PUFA	29.0583	5.268	27.6924	5.556	NS
C18:3n6/C22:4n6	2.8162	2.760	2.9674	2.551	NS

### Correlation matrix

Based on the correlation matrix for parameters of women with CKD and FA concentration in blood plasma, a negative correlation for both sexes was observed between C10:0 and albumin (ALB), and a positive correlation was noted for women with the level of haemoglobin (HGB) and for men with the level of ferritin (FER) (Tables 6 and 7, respectively). There were also frequent correlations between Ca ^2+^ and iPTH in the group of men with CKD, and between Ca^2+^, K^+^, and anthropometric measurements, such as height, weight, BMI, and body surface, in the group of women with CKD. It was also observed that Na^+^ and age did not correlate with any of the analysed FA. In the group of women, C22:4n6n was positively correlated with the duration of the treatment, *C-reactive protein (*CRP), creatinine (CREA), and decreased saturation of transferrin (%TSAT).

**Table 6:** Correlation matrix of the biochemical parameters of women with CKD vs. FFA (selected); *p* < 0.05

Parameter	C10:0 Capric acid	C14:0 Myristic acid	C16:0 Palmitic acid	C16:1 Palmitoleic acid	C18:0 Stearic acid	C18:1n9 ct Oleic acid	C18:2n6c acid	C18:3n6 γ-linoleic acid	C20:5n3 EPA	C22:2 cis-docodienoic acid	C22:4n6 (docosatetraenoate)
Age	0.135	0.090	0,137	0,098	-0,049	−0.193	−0.188	0.226	0.324	−0.080	0.053
Ca [mg/dl]	−0.093	0.310	0,058	0,322	−0,228	0.128	**−0.556**	0.242	**0.760**	0.430	**−0.433**
I PHOS [mg/dl]	−0.171	−0.093	−0,270	−0,210	0,321	0.340	−0.140	−0.105	−0.066	−0.072	−0.423
iPTH [pg/ml]	−0.201	0.027	−0,053	−0,116	0,172	0.236	−0.190	−0.070	−0.072	**0.660**	−0.056
CREA [mg/dl]	0.103	0.163	0,027	0,146	0,026	−0.123	0.207	− 0.230	− 0.167	− 0.212	**0.451**
HGB [g/dl]	**0.482**	0.118	0,268	0,328	− 0,212	− 0.269	− 0.178	0.250	0.298	0.022	0.341
%TSAT [%]	− 0.068	0.150	**0,517**	0,359	**−0,477**	** 0.595**	0.177	0.350	0.316	−0.013	**0.437**
Transferrin	0.428	− 0.092	− 0,166	0,068	− 0,059	0.092	0.090	0.030	0.104	− 0.025	− 0.097
FER [ug/l]	− 0.280	0.059	− 0,095	0,059	− 0,327	0.563	− 0.232	0.328	0.490	**0.827**	− 0.042
ALB [g/l]	**− 0.827**	− 0.067	− 0,033	0,143	0,157	0.301	0.014	0.526	0.212	0.069	−0.594
Na [mEq/L]	−0.064	− 0.355	− 0,415	0,219	− 0,007	−0.397	0.354	−0.035	− 0.303	−0.583	0.405
K [mEq/L]	0.212	−0.318	−0,567	− 0,603	0,414	**− 0.708**	**0.835**	**− 0.746**	**−0.777**	**− 0.743**	0.541
Height [cm]	0.468	**−0.804**	−0,424	− 0,237	0,109	**− 0.760**	**0.821**	− 0.551	**−0.711**	− 0.287	**0.926**
Weight after [kg]	−0.129	**0.388**	0,026	0,258	0,111	**0.448**	**−0.372**	**0.366**	0.183	0.030	−0.237
Treatment duration [months]	−0.115	−0.152	− 0,030	0,006	− 0,127	0.021	0.036	−0.291	− 0.106	0.017	**0.413**
BMI	−0.048	0.355	−0,015	0,335	−0,146	**0.494**	**−0.415**	**0.394**	0.159	0.107	−0.179
Body Surface [m2]	−0.172	**0.408**	0,062	0,181	−0,225	**0.369**	−0.310	**0.358**	0.211	−0.030	−0.290
Kt/V	0.193	−0.347	−0,054	− 0,248	−0,039	**− 0.454**	0.254	− 0.330	−0.176	**− 0.379**	**0.369**
CRP	**0.536**	−0.107	−0,084	− 0,034	0,207	0.064	0.172	−0.345	−0.118	− 0.052	0.091
nPCR [g/kg/day]	−0.024	0.062	0,289	−0,030	−0,017	− 0.017	−0.073	− 0.022	−0.230	− 0.293	0.176
Ca x P [mg2/dl2]	−0.025	0.242	0,189	0,152	−0,168	−0.168	0.084	0.166	−0.011	−0.040	0.352

* Average treatment duration 62.42 months; bold text - statistically significant correlation

**Table 7:** Correlation matrix of the biochemical parameters of men with CKD vs. FFA (selected); *p* < 0.05

Parameter	C10:0 Capric acid	C16:0 Palmitic acid	C16:1 Palmitoleic acid	C18:0 Stearic acid	C18:1n9 ct Oleic acid	C18:1 tans vaccinic acid	C18:3n3 linolenic acid	C20:1 cis11- eico -sanic acid	C22:4n6 (docosatetraenoate)
Age	0.060	0.276	−0,185	−0,122	− 0.304	0.028	− 0.187	−0.170	0.173
Ca [mg/dl]	−0.103	**0.505**	−0,111	0,389	**−0.412**	**−0.423**	− 0.222	−0.057	− 0.311
I PHOS [mg/dl]	−0.304	**− 0.635**	−0,201	− 0,352	0.194	0.218	**0.438**	0.256	0.131
iPTH [pg/ml]	−0.159	**−0.595**	0,017	−0,092	**0.424**	**0.483**	**0.473**	0.251	**0.485**
CREA [mg/dl]	0.190	0.109	−0,106	0,195	0.013	0.063	−0.069	−0.298	**0.725**
HGB [g/dl]	0.261	0.020	−0,075	−0,214	0.197	−0.019	− 0.102	−0.124	0.232
%TSAT [%]	0.075	0.302	−0,074	0,185	−0.333	0.035	0.011	−0.059	0.364
Transferrin	−0.013	0.201	−0,044	0,159	−0.273	**−0.458**	− 0.375	−0.151	− 0.131
FER [ug/l]	**0.433**	−0.034	0,021	−0,241	0.186	0.252	−0.144	−0.002	0.218
ALB [g/l]	**−0.447**	0.064	0,035	0,245	−0.141	−0.165	0.166	−0.191	0.107
Na [mEq/L]	0.057	−0.132	−0,049	0,047	−0.190	0.201	0.131	−0.128	0.364
K [mEq/L]	−0.154	−0.002	− 0,140	0,103	0.254	−0.048	− 0.027	0.115	0.235
Height [cm]	**−0.356**	−0.038	0,007	−0,165	0.137	0.222	0.121	0.125	−0.067
Weight after [kg]	−0.041	−0.090	− 0,110	−0,134	0.102	0.032	−0.055	0.194	−0.117
Treatment duration [months]	−0.044	−0.150	0,141	−0,117	0.174	**0.353**	−0.015	0.099	**0.436**
BMI	0.186	−0.071	−0,141	− 0,015	0.012	− 0.102	−0.109	0.110	−0.030
Body surface [m2]	−0.260	−0.256	− 0,192	−0,254	0.273	0.139	0.274	**0.329**	−0.215
Kt/V	−0.223	−0.050	0,385	−0,061	0.001	0.283	−0.033	−0.154	0.238
CRP	0.287	−0.177	−0,071	− 0,308	0.191	0.105	−0.221	− 0.044	−0.041
nPCR [g/kg/day]	**−0.314**	−0.198	− 0,152	0,070	− 0.102	0.032	0.123	**0.328**	0.005
Ca x P [mg2/dl2]	−0.026	**−0.322**	**− 0,330**	0,094	− 0.112	−0.181	0.088	0.098	−0.154

* Average treatment duration 46,29 months; bold text - statistically significant correlation

In group of women with CKD, the most frequent correlations with biochemical and anthropometric parameters involved the fatty acids C18:1n9, C18:2n6, C18:3n6, and C22:4n6, which are presented in Table 6. In the group of men with CKD, the most common correlations with the analysed parameters related to C10:0, C16:0, and C18:1 t (Table 7).

### Division of groups based on the duration of treatment

**Fig. 1:**
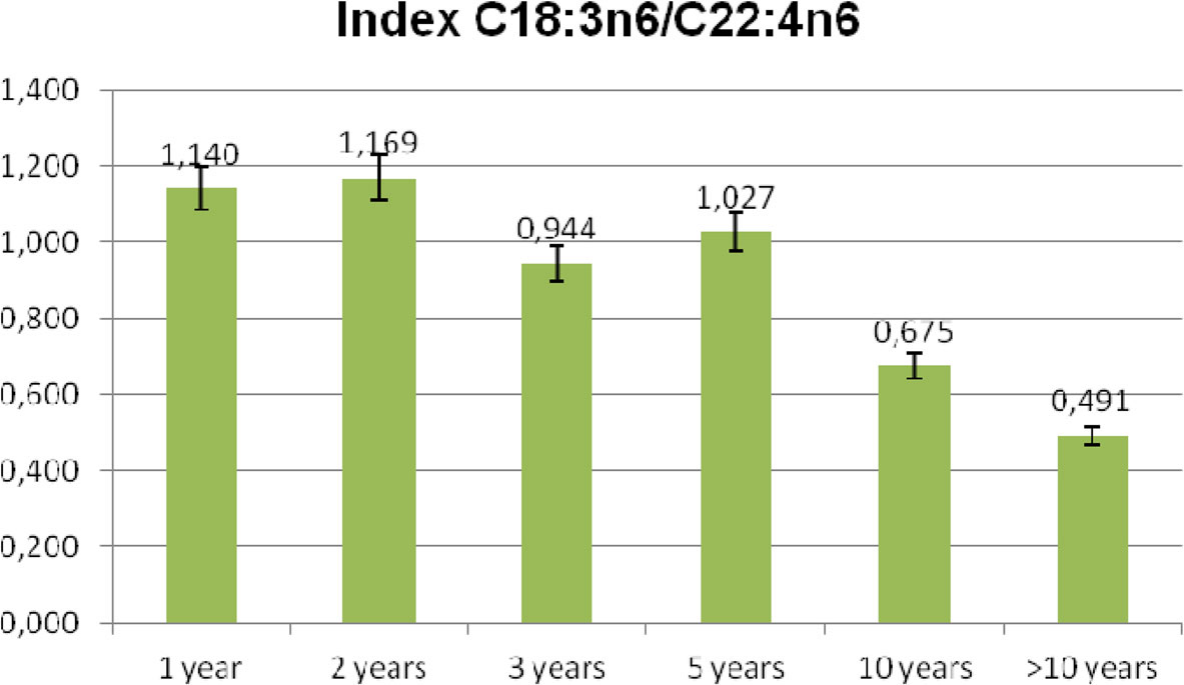
Average values of C18:3n6/C22:4n6 index depending on the duration of the treatment [years]

**Fig. 2:**
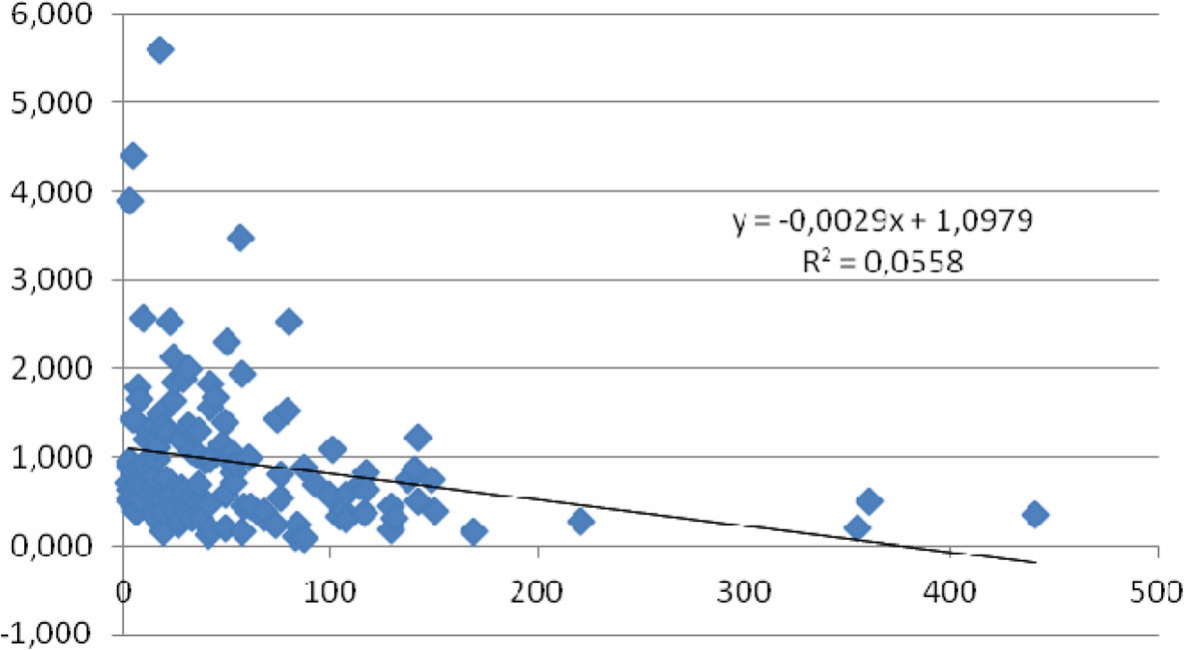
Trend line showing changes in proportions of C18:3n6/C22:4n6 index vs duration of the treatment [months]

As there were no differences in the tendencies of FA concentration changes between the sexes, and observed relations were dependent on the duration of the treatment, all the patients were divided into six groups based on the duration of the treatment. These groups were treated for less than 1 year, up to 2 years, up to 3 years, up to 5 years, up to 10 years, and more than 10 years. Fundamental decreases in the levels of C18:3n6 and C22:4n6 were observed after a period longer than 10 years (Table 8). The calculated index C18:3n6/C22:4n6 in particular groups differed significantly in comparison to the group with the longest period of treatment (Table 8). Moreover, the index C18:3n6/C22:4n6 during the period of the first 5 years remained at a stable level, but later on it began to drastically decrease (Fig. 1). Additionally, by analysing the changes dependent on the duration of the treatment, the trend lines of the decrease in the index (C18:3n6/C22:4n6) were determined (Fig. 2).

**Table 8:** Effect of treatment duration on changes in the C18:3n6/C22:4n6 index

Treatment durtion	Average treatment duration [months]	C18:3n6	C22:4n6	C18:3n6/C22:4n6 index	Index significance > 10 years
1 year *N* = 30	6.100	0.209	3.431	1.140 ± 1.001	0.027717
2 years *N* = 26	17.654	0.211	3.851	1.169 ± 1.064	0.025942
3 years *N* = 24	30.120	0.247	3.431	0.944 ± 0.539	0.015754
5 years N = 24	48.917	0.221	3.206	1.027 ± 0.804	0.022576
10 years N = 24	91.667	0.216	3.866	0.675 ± 0.553	NS
> 10 years *N* = 18	199.71	0.188	3.134	0.491 ± 0.305	–

## Discussion

The key enzyme in fatty acid synthesis—responsible for introducing the first cis double bond in the Δ9 position of the lipotoxic saturated fatty acids stearic acid (C18:0) and palmitic acid (C16:0)—is stearoyl-CoA desaturase − 1 (SCD-1). Such reactions generate less lipotoxic monounsaturated fatty acids (MUFA)—oleic acid (C18: 1n-9) and palmitoyl acid (C16: 1n-7), respectively [18]. In this study, the intensification of these reactions was observed in both groups of men and women with CKD. MUFA, which are the products of SCD catalysed reactions, are used as substrates for the synthesis of phospholipids, triglycerides, and cholesterol esters, and thus may increase the lipid burden on tissues and can initiate insulin resistance in patients [19, 20]. It seems that the intensification of other mechanisms related to SCD-1 activity may occur in the group of women and men with CKD. In our opinion, in the group of women with CKD, the main reason seems to be anthropometric parameters related to fat content, while in the group of men relationships related to calcium and parathyroid hormone levels play a more important role. The adipose tissue synthesises or regulates the production of complement components, in particular C3, in proportion to the amount of adipose tissue that is present. In our studies, we did not examine body fat content, but it is known that women have more than men [21].

Moreover, MUFA also act as mediators of signal transduction, cellular differentiation, and metabolic homeostasis [18, 22]. Therefore, the changes in FA concentration at this stage may increase the diabetic nephropathy (DN) risk through elevated fat content in the liver [7]. Even though oleic acid is the main component of MUFA consumed in the diet, the regulation of SCD-1 gene expression remains governed by hormonal, environmental, and diet factors. For example, a meal high in carbohydrates can quickly induce the SCD-1 gene due to an insulin-dependent increase in the activity of the sterol regulatory element-binding protein (SREBP) -1c and activation of the SCD-1 gene promoter [22]. Other studies show that there exists a SCD regulation on the transcription level, connected to changes in the activity of particular enzymes [23]. These reactions are intensified by oxidative stress, which, due to overproduction of ROS and cellular membrane degradation via lipid peroxidation, may lead to increased levels of myristic acid (C14:0), stearic acid (C18:0), and arachidonic acid (C20:0) [24]. In our study, we observe a slight increase in C14:0 in both sexes, while the other two acids are used in the elongation process. Oxidative processes play a crucial role in damaging renal tubular and renal epithelial cells exposed to ox-LDL activity. Moreover, disrupted transport and FA oxidation, which is accompanied by reduced antioxidative response, damage the structure of podocytes [25]. Podocytes line the outer surface of the basement membrane of the glomerus. Each of the podocytes is linked to more than one arteriole, and each arteriole is covered by several podocytes. Dysregulated lipid metabolism with disrupted metabolism of fatty acids in plasma (FA) is characteristic of metabolic syndrome, obesity, and type 2 diabetes and probably contributes to end-stage renal disease, irrespective of the underlying disease [26].

It was also determined that increased capture of FA by podocytes occurs via increased expression of C36 receptor together with reduced beta-oxidation and, in turn, intracellular lipid accumulation. Therefore, various pathogenetic mechanisms are discussed [25].

Nutritional behaviours have a huge effect on the progression of pathophysiological states. Consumption of food rich in saturated fat accelerates the progression of diseases by the mechanism linked to increased oxidative stress. A study on rats proved that using higher amounts of SFA favours the progression of renal failure [27].

In the cases of all of our patients with CKD (women and men), we observed a decrease in the levels of omega-6 fatty acids and omega-3 fatty acids. Lowering the level of both PUFA families means that this substrate is used in the accompanying inflammatory reaction in patients with CKD. In general, PUFA n-6 promotes inflammation, whereas PUFA n-3 has anti-inflammatory properties [28]. Due to the fact that PUFA n-6 and n-3 compete for the same enzymes for synthesis and metabolism, their presence in tissues is defined by the profile of lipid mediators involved in inflammatory response [29]. A shift in the PUFA n-6/n-3 ratio is currently regarded to contribute to the prevalence of chronic diseases [30]. In fact, many studies have shown the advantageous effect of PUFA n-3 supplementation in various chronic diseases, which was attributed to antioxidative properties of these fatty acids [31]. Interestingly, elevated levels of fatty acids (FA) before a transplantation may be linked to longer survival of kidney transplants. The pre-transplantation level of arachidonic acid (C20:4n6) was independently related to higher survival rates of kidney transplants in multivariate analysis. In one-way analysis, high levels of arachidonic and γ-linolenic FA (C20:0 i C18:3n6) were linked to a higher percentage of patient survival after transplantation [32]. The percentage of GLA C18:3n6 in groups with CKD (men and women) does not change significantly in comparison to the control group because the intake via the diet is similar. However, the levels of C20:3n6 and C20:4n6 (AA) increase due to higher activity of D6D and D5D. Moreover, EPA and DHA also decrease in both groups with CKD as compared to the control group. There was probably the activation of the resolving pathway of anti-inflammatory mediators, synthetizing E and D resolvins, protectins, and maresins. Women are more careful about their diet than men and select pro-healthy products, including those rich in omega-3 fatty acids, which could contribute to a greater extent to the activation of the resolution of inflammation. There is an urgent requirement for other, more natural methods supporting the treatment of the disease.

Correlations between anthropometric and biochemical parameters in men and women with CKD showed various relations resulting from the duration of the treatment of both groups of patients (46.29 and 62.46 months, respectively). The observed relations involve capric acid (C10:0) and are connected to the fact that this acid inhibits receptor activator for nuclear factor κ B ligand (RANKL NF-κB)-dependent osteoclastogenesis, as the level of capric acid decreases together with a longer period of treatment [33]. Albuminuria is a strong, independent prognostic factor for chronic kidney disease, and ferritin in blood plasma, formed due to cell damage, is a well-known marker of inflammation; thus, the correlations are observable [34, 35]. Elongation of the carbon chain in the n-6 family to C22:4n6 was in the male group linked to fewer parameters characterizing organ function (iPTH, CREA, length of treatment) than in the female group (CREA, %TSAT, height, length of treatment, and Kt/V). Elongation of the n-6 family suggests the formation of compounds with mainly pro-inflammatory activity (prostaglandins, leukotrienes) but also of those responsible for resolving inflammation, such as lipoxins [28]. To determine the activation of certain synthesis pathways will require further studies. The ability to form metabolites from the n-3 and n-6 series depends on the activity of Ʌ6-desaturase (D6D), characteristic of the endoplasmic reticulum [36]. This enzyme depends on non-heme iron proteins, and its activity can be measured by determining the concentration ratio GLA/LA. Moreover, the activity of the enzyme depends on the species, age, sex, and the type of diet [37]. As reported by Stawarska et al., a diet low in calories and protein, which is used in CKD, inhibits the activity of this enzyme [38]. Recently, it has been observed that high excretion of both sodium and potassium is linked to increased risk of kidney disease progression in patients with CKD. Multivariate spline regression models suggested a linear correlation between sodium and potassium excretion in urine and the progression of CKD [39]. Therefore, the negative correlations of excreted K^+^ observed in this study confirm the higher risk of CKD progression in patients with lower concentrations of fatty acids.

We should also draw attention to nervonic acid (C24:1), which is a monounsaturated fatty acid belonging to the omega-9 group. It is formed during the elongation of oleic acid (C18:1) to eicosenoic acid (C20:1) and further elongation to nervonic acid [40]. Erucic acid (22:1) consumed with food can also be elongated to nervonic acid in humans [41]. The data coming from patients with chronic kidney disease show that an increasing level of nervonic acid C24:1 is a significant prognostic marker for patient mortality in the 5th stage of the disease [42]. According to Dołęgowska et al., haemodialysis increases the level of nervonic acid in patients with chronic kidney disease [43]. However, in our own studies, the increase in C24:1, though significant in comparison to the control groups, was not as high as for other chronic diseases [43].

Studies on the relation between plasma monounsaturated fatty acids and the prevalence of heart failure have proved that higher levels of the free fatty acids erucic (C22:1) and nervonic (C24:1) acid are connected with heart failure and may indicate the potential cardiotoxicity of long-chain MUFA in humans [44].

There were numerous mechanisms suggested for the contradictory effects of PUFA n-6/n-3, including (a) competition with PUFA n-6 for metabolism and a reduction in the synthesis of eicosanoids originating from PUFA n-6; (b) down-regulation of key enzymes (e.g., COX-2) which synthesise lipid mediators; (c) direct activity on transcription factors (e.g., NF-κB) to inhibit the expression of inflammatory cytokines; and (d) the formation of strong inflammation-resolving compounds (e.g., resolvins, protectins) [45].

When analysing the percentage of particular FA in blood plasma from the patients with CKD, we noticed an increasing amount of C22:4n6 depending on the duration of the disease. Cells metabolize arachidonic acid (AA) to adrenic acid (AdA-C22:4n6) via elongation of the carbon chain. These fatty acids do not compete with each other for esterification to phospholipids. AdA, but not AA, has a tendency to be incorporated into phospholipids containing stearic acid in the sn-1 position [46]. There are significant differences in the cellular uses of AA and AdA by macrophages, since AdA is released only to phosphatidylcholine, whereas AA is released to various molecules of phosphatidylcholine and phosphatidylinositol [47]. The increase in AdA synthesis is probably linked to the synthesis of new dihomo-IsoF compounds, which are metabolites of adrenic acid [47]. It was determined that in coronary arteries, adrenic acid causes endothelium-dependant relaxations mediated by cyclooxygenase and cytochrome P-450 metabolites. The metabolite of AdA: DH-16, 17-EET, activates K (+) in smooth muscles, causing hyperpolarization and relaxation of the endothelium in coronary circulation [48]. Moreover, the metabolites of adrenic acid may function as endogenous derivatives of the endothelium and derivatives hyperpolarizing the zona glomerulosa in the adrenal cortex and thus contribute to the regulation of blood circulation in the adrenal gland [49]. This effect, though so far only showed in animals, may be applicable to people.

## Conclusions

To summarize, the course of inflammation in CKD occurs through decreased medium- and increased long-chain SFA (the synthesis of MUFA); thus, it is similar to that of other chronic diseases. The dominating cascade of changes is the elongation of GLA-C18:3n6 into DGLA-C20:3n6 and AA-C20:4n6. In the course of CKD progression, along with worsened anthropometric parameters and increased excretion of potassium, the activity of Ʌ6-desaturase probably decreases, which reduces the synthesis of EPA and DHA and disables the production of pro-resolving derivatives. With longer duration of the disease, the synthesis of AdA-C22:4n6 increases, and the C18:3n6/C22:4n6 ratio drastically drops after 5 years of disease duration. This parameter can be used to diagnose disease progression. The sample size should be further expanded in future studies, and the influence of cardiovascular and cerebrovascular disease, amyloidosis, multiple myeloma, and other risk factors should be excluded so as to reduce the potential impact on the target value.

## Data Availability

Please contact author for data requests.

## Abbreviations

AdA-C22:4n6: adrenic acid
ALB: albumin
CKD: chronic kidney disease
CREA: creatinine
CRP: *C-reactive protein*
CVD: cardiovascular diseases
C18:3n6/C22:4n6: gamma linoleic acid/ docosatetraenoate
DN: diabetic nephropathy
DHA: docosahexaenoic acid
EPA: eicosapentanoic acid
FA: fatty acid
FER: ferritin
GFR: glomerular filtration rate
GLA: gamma- linolenic acid
HGB: haemoglobin
iPTH: parathyroid hormone
Kt/V: dialysis adequacy
LA: Linoleic *acid*
MUFA: monounsaturated fatty acids
NAFLD: non-alcoholic fatty liver disease
PUFA: poly-unsaturated fatty acids
SFA: saturated fatty acids
T2DM: type 2 diabetes
